# Assessing the long-term persistence of SARS-CoV-2 in Guinea: insights from post-epidemic sentinel syndromic surveillance data

**DOI:** 10.3389/fepid.2025.1636286

**Published:** 2025-09-25

**Authors:** Kadio Jean Jacques Olivier Kadio, Thibaut Armel Chérif Gnimadi, Emilande Guichet, Castro Gbêmemali Hounmenou, Abdoul Karim Soumah, Haby Diallo, Amadou Camara, Saidouba Chérif Camara, Marie Rose Sandouno, Salifou Talassone Bangoura, Maladho Diaby, Vincent Richard, Julien Poublan, Sidikiba Sidibé, Alexandre Delamou, Alioune Camara, Alpha Kabinet Kéita, Eric Delaporte, Abdoulaye Touré, Afroscreen Team

**Affiliations:** ^1^ Centre de Recherche et de Formation en Infectiologie de Guinée (CERFIG), Gamal Abdel Nasser University of Conakry (UGANC), Conakry, Guinea; ^2^ Chaire de Santé Publique et Politique Pharmaceutique, Department des Sciences Pharmaceutiques et Biologiques, Faculté des Sciences et Techniques de la Santé (FSTS), Gamal Abdel Nasser University of Conakry (UGANC), Conakry, Guinea; ^3^ Institut for Research and Development, IRD/UMI233/INSERMU1175, Montpellier University, Montpellier, France; ^4^ Head of Programs for Promotion (P4P) Division, International Affair Department, Institut Pasteur, Paris, France; ^5^ Bordeaux Population Health Research Center (BPH), Global Health in the Global South (GHiGS), INSERM (U1219)—IRD (EMR 271), Université de Bordeaux, Bordeaux, France; ^6^ Chair of Public Health, Department of Medical Sciences, Faculty of Health Sciences and Techniques, Gamal Abdel Nasser University of Conakry, Conakry, Guinea; ^7^ African Centre of Excellence for the Prevention and Control of Communicable Diseases (CEA-PCMT), Gamal Abdel Nasser University of Conakry, Conakry, Guinea; ^8^ National Malaria Control Program, Ministry of Health and Public Hygiene, Conakry, Guinea

**Keywords:** SARS-CoV-2, Omicron, AFROSCREEN, reproduction number, sentinel syndromic surveillance, genomic surveillance, Guinea normal (Web)

## Abstract

**Background:**

In December 2019, the world experienced one of the significant health crises of the 21st century with the emergence and rapid spread of the potentially fatal 2019 coronavirus (COVID-19). In this context, sentinel surveillance of SARS-CoV-2 variants was conducted in Conakry. Here we report the first data on reproduction numbers and risk factors during the Omicron post-epidemic period in Guinea.

**Methods:**

A sentinel syndromic and genomic surveillance study was conducted on suspected patients from October 2022 to July 2024 at healthcare facilities in Conakry. Individual data and nasopharyngeal swabs were collected and sent to the Centre de Recherche et de Formation en Infectiologie de Guinée (CERFIG) laboratory for screening and sequencing by next-generation sequencing (NGS). The effective reproduction number (Rt) were estimated using EpiEstim to assess the transmission potential of the Omicron variant. Generalized linear models based on the binomial distribution were employed to analyze factors associated with SARS-CoV-2 positivity, following the identification of primary risk factors using Bayesian model averaging and the Data balancing algorithm using propensity score matching.

**Results:**

Data from 1174 patients with suspected cases with a median age of 31 years (IQR: 20–51), were analyzed. The overall COVID-19 positivity rate was 11.8%. The global effective reproduction number (Rt) was 2.08 [95% CI: 0.35–5.81]. Only ageusia [AOR = 2.0; 95% CI (1.1–3.6)] was independently associated with SARS-CoV-2 test positivity.

**Conclusion:**

SARS-CoV-2 is still circulating in Guinea, with a high positivity rate and a high number of effective reproductions in this post-epidemic period in our country. The associated factors and the circulation of variants with a diversity of circulating strains suggest the need to strengthen genomic and epidemiological surveillance, with the support of all those involved in the response to COVID-19, to ensure continuity of alerts and decision-making for public health.

## Introduction

In December 2019, the world faced one of the most significant health crises of the 21st century with the emergence and rapid spread of the potentially fatal coronavirus 2019 (COVID-19). The infection, caused by severe acute respiratory syndrome coronavirus 2 (SARS-CoV-2), was first reported in Wuhan, Hubei province, China (1). At the onset of the disease, in China the basic reproduction number (R_0_) was estimated to range between 2.24 (95% CI: 1.96–2.55) and 3.58 (95% CI: 2.89–4.39) (2, 3) with the mean incubation period of 6.4 days (range: 2.1–11.1 days) and evidence of potential asymptomatic transmission (2, 4). In Africa, an analysis of data from 2020 estimated a reproduction rate of 2.02 ± 0.7, ranging from 1.12 to 3.64 (5). By late 2021, following the emergence of the Omicron variant, multiple studies estimated the basic (R0) and effective (Rt) reproduction rates for this variant and its early sub-variants. An analysis of 15 studies covering Europe, America, Asia, and three studies from South Africa (the only African country included), reported mean numbers of basic (R0) and effective (Rt) reproduction values for the Omicron variant at 9.5 (range: 5.5–24) and 3.4 (range: 0.88–9.4) respectively (6). Since the start of the pandemic, the attack rate of COVID-19 has increased, with regional variations observed in African countries (7). In addition, as the pandemic evolves, the SARS-CoV-2 virus, like any pathogen, mutates over time (8). These mutations are manifested by the increased transmissibility of the virus, disease severity, and escape of neutralizing antibodies, and are classified as variants of concern (VOC) (9). These variants typically result in a moderate clinical presentation of the disease, with symptoms resolving within 2–6 weeks, except for the Delta variant (10). Common symptoms of COVID-19 include myalgia, nasal symptoms, headache, fever, asthenia, dry cough, difficulty breathing, sore throat, chest pain, runny nose, and diarrhea, loss of taste or smell (11–14). Several studies have also reported factors associated with COVID-19 positivity. Studies in Europe (15) and Africa (16, 17) have identified numerous factors associated with SARS-CoV-2 positivity, including contact with a confirmed case, the presence of one or more household members, typical SARS-CoV-2 symptoms, male gender, fatigue, fever, cough, headache and respiratory problems. The variation in reproduction numbers, symptoms, and associated factors across different geographical areas highlights the similarities in symptoms found in most infectious diseases and the multiplicity of presumed associated factors. This suggests the need for systematic screening for this disease in healthcare settings. Understanding the factors associated with positivity is essential for improving surveillance and thus, effectively reduce transmission of COVID-19, reviewing screening strategies, strengthening community communication, and enhancing management efforts.

Following the global outbreak of COVID-19, particularly in Africa, the surveillance of respiratory diseases has gained increasing attention in several Sub-Saharan African countries (18, 19). Consequently, international public health institutions have recognized the necessity for joint coordination in the surveillance of these severe acute respiratory diseases (18, 19).

In Guinea, since the official declaration of the first cases in March 2020 by the Ministry of Health and Public Hygiene, Conakry has remained the epicenter of the COVID-19 pandemic, accounting for over 80% of cases and low vaccination coverage of 28% in February 2023 according to the Agence Nationale de Sécurité Sanitaire (ANSS) (20). Several public health institutions are involved in the epidemiological surveillance of severe acute respiratory infections (ARIs) in the country, including the Centre de Recherche et de Formation en Infectiologie de Guinée (CERFIG).

With this in mind, CERFIG has established surveillance of COVID-19 variants in sentinel sites. We report here the first data on reproduction numbers and risk factors in the post-epidemic period.

## Methods

### Study setting, design, period and population

The study was carried out in Conakry, the capital and largest city of the Republic of Guinea, with an estimated population of 2,095,705 in 2022 (21). It is a peninsula covering an area of around 308 km^2^, subdivided into five municipality: Kaloum, Matam, Dixinn, Ratoma and Matoto. There are three national hospitals, six communal medical centers, twenty-seven polyclinics and three hundred and ten clinics or medical practices spread throughout the capital's five communes (22). As part of this study, five health establishments (the Pneumology Department of the Hôpital National Ignace Deen, the Emergency Department of the Hôpital National Conakry, the Centre de Traitement des Epidémies de Nongo (CTEpi), the Centre Médical Municipal de Ratoma and the Formation Sanitaire de Koulewony) were identified as sentinel sites, with the support of the Guinean Ministry of Health and Public Hygiene. These sites were chosen on the basis of their activities in the response to COVID-19, but also for their experience in influenza surveillance for others. We conducted a study based on sentinel syndromic surveillance and genomic surveillance was carried out among outpatients and inpatients during the period from October 2022 to July 2024. The study population included all patients attending the above-mentioned sentinel sites who were identified as suspected cases of COVID-19 according to the WHO definition (23). A suspected case was defined as a patient presenting with one or more of the following symptoms (fever, cough, runny nose, dyspnea, sore throat, as well as any other respiratory symptoms), during a visit to one of the sentinel sites. A confirmed case was defined as an individual with a positive PCR test result (cycle threshold (Ct) value < 40.0). This surveillance was carried out as part of the AFROSCREEN project, aimed at strengthening surveillance of SARS-CoV-2 circulation in 13 African countries, including the Republic of Guinea. It was set up during a period of closure of virtually all screening and management sites for suspected and confirmed cases of COVID-19 in Conakry and throughout the country.

### Data collection

Data were collected using a standardized individual survey form. The collected data included socio-demographic characteristics (age, sex, occupation, marital status, level of education, and number of people living with the case), clinical information (fever, cough, dyspnea or respiratory distress, sore throat, cold, headache, agueusia, anosmia, asthenia, muscle soreness etc.), medical history (obesity, hypertension, diabetes, HIV, TB, asthma), exposure and vaccination status, and biological data (PCR result, variant, and sub-variant). In accordance with Centre de Recherche et de Formation en Infectiologie de Guinée (CERFIG) surveillance procedures and logistics, nasopharyngeal swabs were taken from suspected cases between 8: 00 am and 12: 00 am GMT at sentinel sites during daily consultations, after obtaining free and informed consent. They were then stored in coolers containing cold accumulators and sent for screening and Next-Generation Sequencing (NGS) to the virology laboratory of the Centre de Recherche et de Formation en Infectiologie de Guinée (CERFIG).

### Laboratory analysis

For nasopharyngeal swabs, viral RNA was extracted manually using the RunMei kit and amplified on the Bio-Rad CFX96 PCR machine (Bio-Rad Laboratories S.r.l). Molecular tests confirmed positivity for SARS-CoV-2 infection if a cycle threshold (Ct) <40.0 was found and negativity if the cycle threshold (Ct) value was ≥40.0 or when there was no amplification. To characterize the viral strain, virus genome was generated using CovidSeq protocol (Illumina Inc, USA) on an Illumina ISeq100 platform. Raw data were analyzed using an in-house pipeline developed for the AFROSCREEN sequencing network (https://forge.ird.fr/transvihmi/nfernandez/GeVarLi) for quality control, alignment, variant calling, mapping to reference genome, and consensus sequence generation.

### Data analysis

Quantitative variables were expressed as median and interquartile range (IQR), since normality was not respected (*p* < 0.05 after Shapiro–Wilk test). Qualitative variables were presented as absolute frequency and percentage. The effective reproduction number (Rt) was calculated using the “EpiEsptim” package of the R sofware taking into account the incidence of COVID-19 over the monitoring period and the overall mean generation time of COVID-19 of 4.7 days with a standard deviation of 2.9 (24, 25). Generalized linear models based on the binomial distribution in multivariate analysis were used to analyze the factors associated with positivity to SARS-CoV-2 infection, following the identification of the main risk factors using the Bayesian model averaging approach developed by Kass and Raftery (26, 27). The stepwise procedure with various stopping rules or the selection method in bivariate analysis for the selection of independent variables in the multivariate analysis initially chosen was abandoned in the face of certain limitations due to the relatively large number of variables to be analyzed, in particular with a small sample size and a low event rate (27, 28). As for the selection method in bivariate analysis, it does not adequately control for confounding or intercorrelations between independent variables, inducing bias in the estimation of the effects of a risk factor (28). As for the stepwise procedure, by excluding non-significant variables, this approach underestimates the uncertainty associated with the model, and implicitly assumes that the final model is “optimal”, which is not necessarily the case (27). The Bayesian model averaging approach used for a more appropriate selection of variables therefore takes into account the uncertainty that may be present in the final model, by integrating several models into the analysis, thus providing a more robust estimate of the effects of the variables on the event of interest (28). It consists in calculating an average of the posterior distributions of the identified models, weighted by their posterior model probabilities. The statistical performance criteria used to select the best Bayesian model are: (i) Posterior model probabilities (PMPs): These probabilities assess the credibility of each model in relation to the others. Models with PMPs within a factor of 20 of the most probable models are considered relevant for the analysis and (ii) Occam's Window: This method is used to include only those models that meet a certain probability threshold in the teaching process, thus contributing to optimal model selection without overfitting (29). The nearest-neighbor matching method (on the default propensity score) was used to balance the classes using the MatchIt” package in the software (Supplementary Material 1A,A bis). It performs matching, subset selection and sub-classification with the aim of creating groups between the minority class and the majority class that are balanced according to the covariates included (30). Multicollinearity was also assessed using correlation matrices to ensure that the maximum values of the coefficients did not exceed 0.8 in absolute value, as well as with the use of the ‘‘performance’’ function from the R package, which provides indicators such as the Variance Inflation Factor (VIF). Finally, the balanced data set obtained was subjected to multivariate analysis. Generalized linear models based on the binominal distribution with different link functions (probit, logit, clog, cauchit) were tested. Parsimony was assessed using the anova function from the R package, and the model with the best fit to the data was selected based on an Akaike Information Criterion (AIC) value below one and a delta AIC less than two (Supplementary Material 2A,B). Adjusted odds ratios (AOR) and their 95% confidence intervals were calculated. A *p*-value < 0.05 was considered significant. REDCap 12.5.9 software was used for data entry and R Studio 4.5.1 (31) for statistical analysis.

### Ethical approval

The study protocol was approved by the National Health Research Ethics Committee (CNERS) of Guinea (N° 199/CNERS/23). Free and informed consent was obtained from patients prior to data collection, and the information was collected anonymously and confidentially.

## Results

### Socio-demographic characteristics of suspected cases

Socio-demographics characteristics of the participants are presented in Table 1. From October 2022 to July 2024, data from 1,174 suspected cases with a median age of 31 years (IQR: 20–51) were analyzed. Most participants were married (47%) and had completed higher education (31%) or secondary education (30%). Additionally, 22% had no formal education. The median number of people living with the suspected cases was 5 (IQR: 4–7).

**Table 1:** Characteristics of suspected COVID-19 cases received at COVID-19 sentinel surveillance sites in Conakry (October 2022–July 2024).

Characteristic	N or Me (IQR)	%
Age (years)		
1–17	212	18
18–24	205	17
25–40	356	30
40+	401	34
Median (IQR)	31 (20, 51)	
Sex		
Female	599	51
Male	575	49
Marital status		
Divorced	14	1.2
Married	556	47
Single	517	44
Widow(er)	87	7.4
Surroundings	5.00 (4.00, 7.00)	
Instruction		
None	253	22
Literate	35	3.0
Primary	176	15
Secondary	348	30
Higher	362	31

### Clinical characteristics of suspected cases

Clinical symptoms, medical story, vaccination and exposure status of suspected cases are described in Table 2. Most common symptoms were cough (83%), cold (75%), headache (69%), fever (67%) and asthenia (57%). In addition, other symptoms include sore throat (46%), muscle soreness (37%), dyspnea (33%), ageusia (26%), anosmia (20%) and arthralgia (19%). Of all the suspects with a medical history (35%), the underlying or previous illnesses were tuberculosis (13%), hypertension (12%), diabetes (4.2%), HIV (3.1%) and sinusitis (2.9%). Nearly three-quarters of the participants had a moderate level of disease severity, and 29% were hospitalized. Additionally, 48% of the participants reported to have been vaccinated against COVID-19. The vaccines most frequently received were Sinovac® (28%) and Johnson-Johnson® (18%). In terms of exposure characteristics, 64% reported to have attended mass gatherings and 16% to have been exposed to someone with similar symptoms in the 14 days before the onset of symptoms. In addition, 4.7% had been in contact with suspected or confirmed cases and 4.8% had traveled in the 14 days before the onset of symptoms.

**Table 2:** Clinical symptoms, medical story, vaccination and exposure status of suspected cases received at COVID-19 sentinel surveillance sites in Conakry, October 2022–July 2024.

Characteristics	*N*	%
Clinical symptoms		
Sign	1,136	97
Cough	977	83
Cold	879	75
Headache	805	69
Fever	781	67
Asthenia	675	57
Sore throat	537	46
Muscle soreness	429	37
Dyspnea	388	33
Agueusia	310	26
Anosmia	237	20
Arthralgia	218	19
Vomiting	49	4.2
Abdominal pain	47	4.0
Medical history	406	35
TB	150	13
Hypertension	138	12
Diabetes	48	4.1
HIV	37	3.2
Sinusitis	34	2.9
Obesity	16	1.4
Degree disease severity		
Moderate	874	74
Severe	101	8.6
Simple	199	17
Hospitalization	345	29
Vaccination	569	48
SinoVac/Sinopharm	332	28
Johnson-Johnson	217	18
Exposure status		
Travel	56	4.8
Contact of suspected or confirmed case	55	4.7
Gathering	746	64
Exposed to similar symptom	192	16

### Positivity and effective reproduction numbers

**Figure 1:**
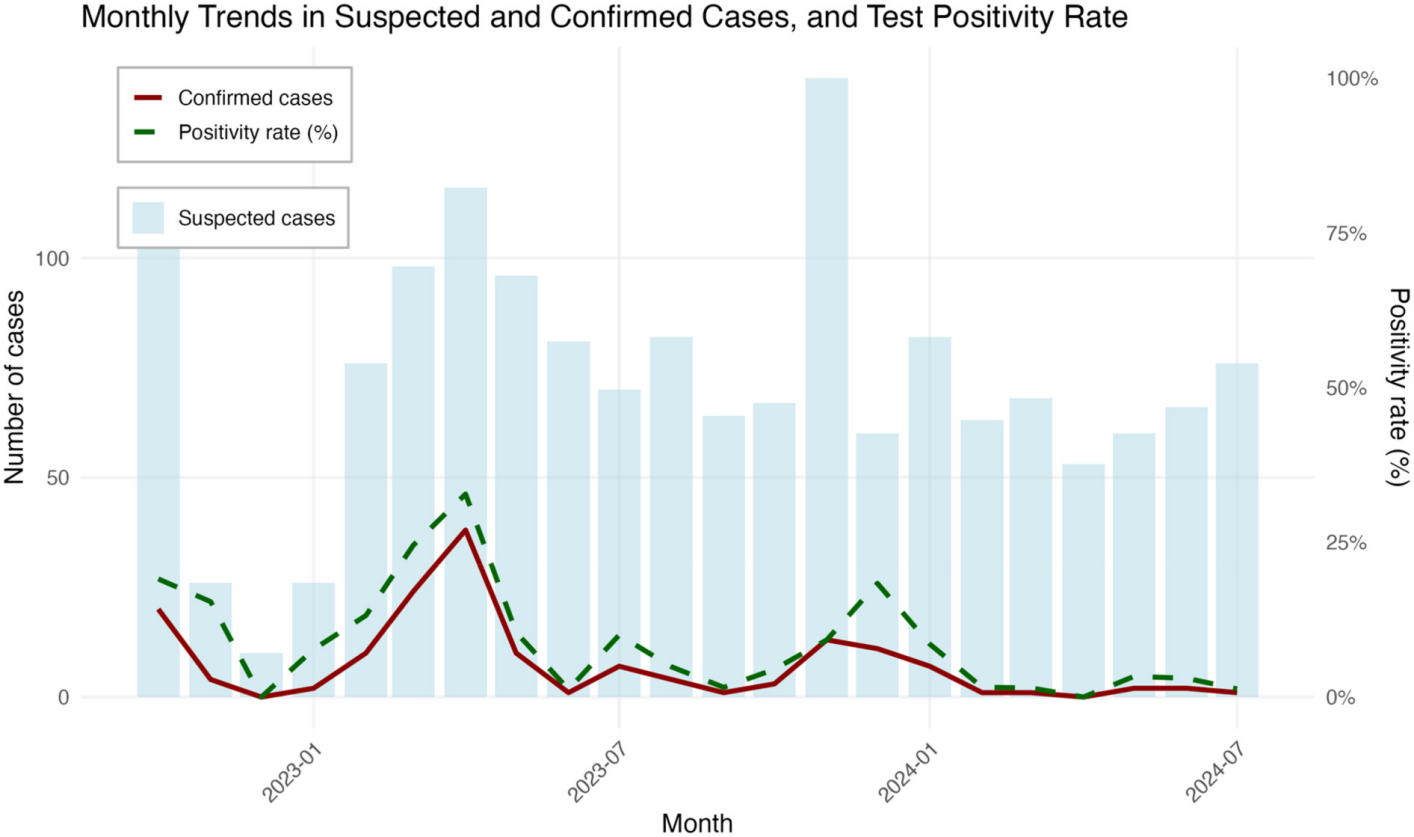
Epidemiological curves and monthly trends during sentinel surveillance for SARS-CoV-2 in Conakry, October 2022–July 2024.

The overall positivity rate for COVID-19 was 11.8% (139/1,174). The highest peaks in SARS-CoV-2 positivity were observed in April (32.75%) and March 2023 (24.48%). Positivity rates exceeding 10% were also reported in October 2022 (19%), December 2023 (18.3%), November 2023 (15.38), and February 2023 (13.15%) (Figure 1). Furthermore, of the 139 positives, 7.4% of sequences were identified as the Omicron variant. The most frequently identified sub-lineages wereXBB.1.5 (49.4%), XBB.1 (12.3%), BQ.1.1 (6.2%), BA.2.86 (6.2%), BA.2 (4.9%), and XBB.1.4 (4.9%).

**Figure 2:**
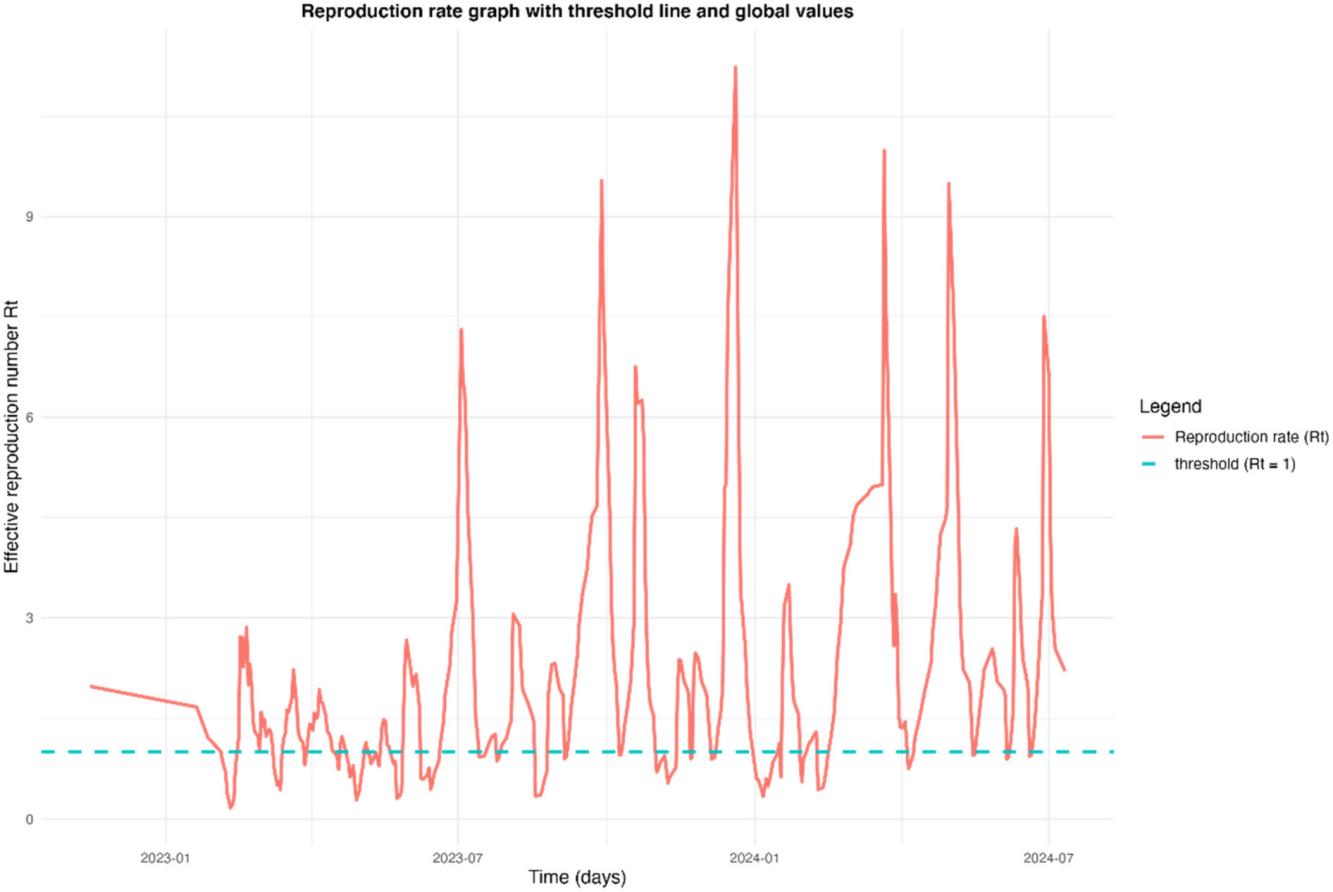
Time-varying effective reproduction number (Rt) of COVID-19 cases in Conakry, Guinea, from October 2022 to July 2024.

The effective reproduction number (Rt) of syndromic surveillance was estimated at 2.08 (95% CI: 0.35–5.85) (Figure 2). The graph obtained would have revealed that there were three transmission periods with effective reproduction numbers (Rt) above 1. Thus, the periods of high epidemic peaks would have been observed between July and December 2023 and between February and July 2024. However, despite the high peaks observed during these periods, there were also periods of stabilization (Rt ≈ 1) and decline (Rt < 1). In addition, in July 2024, the epidemic was still present, with an effective reproduction number of around 2 (Rt = 2.08).

### Risk factors of SARS-CoV-2 infection

**Figure 3:**
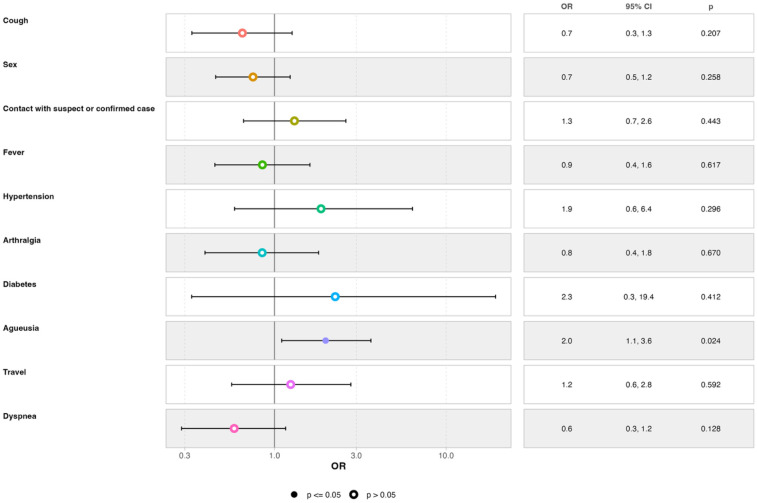
Multivariate regression of factors associated with COVID-19 positivity in suspected cases at sentinel surveillance sites in Conakry, Guinea, October 2022 - July 2024.

Multivariable regression analysis revealed that only ageusia (AOR = 2.0; 95% CI [1.1–3.6]) was associated with a higher likelihood of testing positive among suspected cases (Figure 3).

## Discussion

In a context where the number of screening sites in the country has been reduced by more than 90%, and as part of the support for surveillance of COVID-19, including its variants, we analyzed transmission rates and factors associated with SARS-CoV-2 infection among suspected cases in health facilities in Conakry.

The positive rate for SARS-CoV-2 infection found in suspected cases exceeded 10% in Conakry. For comparison, rate were reported at 6.4% in four sub-Saharan African countries (Côte d'Ivoire, Burkina Faso, Central African Republic and Madagascar), ranging from 4.0% to 16.6% (32).

A sentinel syndromic surveillance study in Malawi from July 2020 to April 2022 revealed a SARS-CoV-2 positivity rate of 11.5% (33). In Madagascar, a higher positivity rate of 24.5% was recorded between March 2020 and May 2022 (34). In Ethiopia, authors reported a 9.2% positivity rate at national sentinel surveillance sites for severe acute respiratory infections (SARI) and influenza-like illness (ILI) over an 18-month period (January 2021 to June 2022) (35). Similarly, a study in Kenya conducted across eight sentinel sites between April 2020 and March 2022 reported a positivity rate of 10.7% (36). In Uganda, a study from January to December 2022 found a positivity rate of 7.7% (37).

Our results indicated a slightly higher positivity rate during the surveillance period, occurring against the backdrop of the May 2023 declaration of the end of the international emergency (38). This led to reduced screening and barrier measures across the country, including during national and international travel. Variations in positivity rates can be attributed to differences in sample sizes, surveillance durations, study populations, and local sample collection strategies.

Additionally, our study identified the first case of the Omicron variant in Guinea. All sequences obtained were of the Omicron variant, with a high frequency of the XBB.1.5 sub-lineage. This sub-variant, first identified in August 2022, had been predominant globally since February 2024 and was noted for being more transmissible than its XBB.1 counterpart (39). The sub-variants found in our study align with those identified elsewhere during the pandemic, including their emergence periods (40, 41). In addition, the variations of effective reproduction number (Rt) revealed a complex epidemic dynamic, characterized by periods of intense transmission and phases of stabilization or decline. This would suggest that several epidemic waves followed one another during these periods, but also that the virus was circulating actively, probably encouraged by factors such as social behavior, environmental conditions or insufficient collective immunity. The persistence of a high Rt (Rt = 2.08) in July 2024 gives cause for concern. This suggests that the epidemic was not yet under control at that date, with the potential for transmission still significant. This figure indicates that each infectious case generated an average of two others, which could have led to a new wave if additional measures were not taken. This situation could be explained by the emergence of new variants, a drop in adherence to health measures, or insufficient vaccination coverage. In addition, although progress was made during the warm periods of the pandemic, the introduction of this surveillance system alerted public health players to the probable emergence of new strains and the need for ongoing surveillance to prevent future epidemic waves and protect public health. Similar data were reported in Mali and Senegal, with reproduction rates of 3.98 (90% CI: 3.61–4.43) and 3.78 (90% CI: 3.16–4.10), respectively (42). A study analyzing data for the period March to May 2020, generated by the COVID-19 Data Repository by the Center for Systems Science and Engineering (CSSE) at the Johns Hopkins University, revealed that an estimated basic reproduction number of 1.61 (1.46, 1.77) for Guinea (43).

These results highlight the rapid circulation of COVID-19 in Conakry and other African cities during this period, despite vaccination campaigns and a global decline in cases. A seroprevalence survey conducted in June 2022 in Conakry indicated an overall seroprevalence of IgG against the spike and core proteins of SARS-CoV-2 at 71.57% (44). This survey suggested that nearly the entire population of Conakry had been in contact with the virus, which may support the Rt values observed during the surveillance period. Furthermore, it suggests poor screening and underreporting of cases at the national level, abandonment of preventive measures (such as social distancing and participation in mass gatherings), and potential waning of vaccine immunity. The WHO reported in its latest COVID-19 global risk assessment in June 2024 that positivity rates for SARS-CoV-2 infection in sentinel sites and wastewater surveillance indicate high circulation worldwide (45), corroborating the results from our surveillance.

The results of the multivariate analysis revealed that the presence of ageusia would increase the likelihood of testing positive for SARS-CoV-2 infection. Previous studies have indicated that factors associated with COVID-19 in Africa include fever, cough, headache, respiratory problems, and age ≥60 years are the factors associated with COVID-19 in Africa (16, 46). Given the combination of these factors, it is important to consider other pathologies as well. Data from studies conducted in some European countries show that anosmia, ageusia, fever, breathlessness, and cough were strongly associated with test positivity. The association between symptoms and test status varied based on the duration of illness, timing of testing, broader testing criteria, and context (by country and testing platform) (47). In Serbia, during a period of Omicron circulation, authors found that hospitalized, elderly, unvaccinated, and previously infected patients, as well as smokers, were more likely to test positive for SARS-CoV-2 (48). Other studies have noted associations between SARS-CoV-2 positivity and sensory deficiencies, severe symptoms, loss of smell, loss of taste, cardiovascular disease, neuropsychiatric disease, and endocrine disease (49). Hunter et al. reported that factors such as mask-wearing habits, foreign travel history, household size, employment status, and contact with specific age groups contributed to the risk of SARS-CoV-2 positivity (50). The variations in associated factors across these studies may be attributed to differences in data collection methods, statistical approaches, and sample sizes.

This study has certain limitations. Firstly, symptom and vaccination status data were collected declaratively, and the cross-sectional nature of the study may lead to reporting bias. The sample only included individuals who visited the sentinel sites, limiting the generalizability of the results to the entire population of Conakry. Positivity rate of 11.8% could indicate incomplete case detection, particularly among asymptomatic or minimally symptomatic individuals. Secondly, although the estimated effective reproduction rate (Rt = 2.08) reflects active transmission of the virus, it could be amplified by delays in reporting or changes in screening policies. Furthermore, the results are based on global data, without explicit consideration of contextual or regional disparities that could affect epidemic dynamics.

Nevertheless, this study raises significant issues. It is one of the first to focus on calculating the effective reproduction numbers of the Omicron variant, as well as the risk factors for COVID-19 in the post-epidemic period. Additionally, it underscores the ongoing and likely large-scale circulation of SARS-CoV-2 and its variants in Conakry. Surveillance of epidemic diseases in the Guinean healthcare system dates back to the colonial period, characterized by the intervention of mobile teams to combat endemic diseases and the selective establishment of hospitals in major cities (48). This surveillance system, centered on primary health care (PHC), has evolved over time and has faced a resurgence of epidemic diseases (Ebola, Marburg, Lassa, COVID-19, etc.) since 2014, within a context marked by numerous vertical projects and programs (51).

The sentinel surveillance conducted in this study represents the only syndromic surveillance of COVID-19 currently in effect in Guinea during this post-epidemic period. This aligns with the latest WHO guidelines, which recommend collaborative surveillance of COVID-19 to inform situational awareness, risk assessment, and the detection of significant changes in virus characteristics, transmission, disease severity, and population immunity. It is also essential to continue making COVID-19-related data (including mortality and morbidity statistics, SARS-CoV-2 genetic sequences, and metadata) available through open sources (45).

The data collection strategy employed for this surveillance involved telephone calls and face-to-face interviews during consultation visits over a 22-month period. A notable strength of this work is the use of the Bayesian model-averaging approach for identifying factors associated with SARS-CoV-2 positivity, which provides a more robust estimate of the effects of variables on the event of interest by integrating multiple models into the analysis (28). This method selects only those models that meet a certain probability threshold, aiding optimal model selection without overfitting. Additionally, multivariate logistic regression was applied using a sub-sampling technique to address class imbalance in the datasets (48).

## Conclusion

SARS-CoV-2 continues to circulate in Guinea, with high positivity rates and effective reproduction numbers in excess of 1 in the post-epidemic period. The disease remains poorly understood due to the numerous mutations of the virus, which can contribute to increased transmissibility and severity of illness. Factors independently associated with test positive SARS-CoV-2 infection was ageusia. This study underscores the persistence of COVID-19 cases and emphasizes the necessity of ongoing variant monitoring, particularly in light of the observed reduction in COVID-19 cases globally. The diversity of circulating strains highlights the urgent need to enhance genomic and epidemiological surveillance. Implementing effective screening strategies in healthcare facilities, along with preventive measures, is essential. Collaboration among all stakeholders involved in the COVID-19 response is crucial to ensure continuous alertness, informed public health decision-making, and to mitigate the risk of potential epidemic outbreaks.

## Data Availability

The raw data supporting the conclusions of this article will be made available by the authors, without undue reservation.
