# Supplementary material for: Assessing the long-term persistence of SARS-CoV-2 in Guinea: insights from post-epidemic sentinel syndromic surveillance data
Source: Front Epidemiol. 2025 Sep 25;5:1636286. doi: 10.3389/fepid.2025.1636286 (PMC12507806; doi:10.3389/fepid.2025.1636286)
Supplement: Supplementary file 1 [file Table1.docx]

**Supplemental Material 1A** Balance of covariates before and after propensity

score matching

| Balance of covariates before/after matching | | | |
| --- | --- | --- | --- |
| **Covariates** | **Type** | **Diff.Un** | **Diff.Adj** |
| distance | Distance | 0.9657993 | 0.2881362 |
| Sex | Binary | 0.0169673 | -0.0647482 |
| Age | Contin. | 0.2047719 | 0.0157015 |
| Surroundings | Contin. | 0.1457650 | 0.0381933 |
| Fever | Binary | 0.0288117 | -0.0575540 |
| Cough | Binary | -0.1034373 | -0.0791367 |
| Dyspnea | Binary | -0.1137455 | -0.0575540 |
| Sore throat | Binary | 0.1339937 | 0.0359712 |
| Cold | Binary | -0.0250721 | 0.0215827 |
| Agueusia | Binary | 0.1982692 | 0.0503597 |
| Anosmia | Binary | 0.1545546 | 0.0287770 |
| Asthenia | Binary | 0.0006603 | 0.0000000 |
| Headache | Binary | 0.0137838 | 0.0143885 |
| Muscle soren | Binary | 0.1404164 | -0.0143885 |
| Vomit | Binary | -0.0065409 | -0.0071942 |
| Diarrhea | Binary | -0.0068675 | 0.0071942 |
| Abdominal pain | Binary | -0.0209293 | 0.0071942 |
| Arthralgia | Binary | -0.0637403 | -0.0287770 |
| Obesity | Binary | -0.0072985 | 0.0071942 |
| Diabetes | Binary | -0.0137351 | 0.0143885 |
| Hypertension | Binary | -0.0517291 | 0.0287770 |
| Asthma | Binary | 0.0094533 | 0.0000000 |
| TB | Binary | -0.0551628 | -0.0215827 |
| HIV | Binary | -0.0194279 | 0.0000000 |
| Chronic respiratory | Binary | -0.0068675 | 0.0000000 |
| Vaccination Covid-19 | Binary | 0.2418031 | 0.0071942 |
| Travel | Binary | 0.0927814 | 0.0359712 |
| Contctact with suspect or confirmed case | Binary | 0.1590310 | 0.0359712 |
| Gathering | Binary | -0.0026553 | 0.0000000 |
| Exposed to similar symptoms | Binary | 0.2388350 | 0.0143885 |
| Hospitalisation | Binary | 0.0991694 | 0.0000000 |

Following matching, all covariates exhibited adjusted standardized mean differences (Diff.Adj) below 0.1, suggesting adequate balance between treatment groups and effective reduction of confounding.

**Supplemental Material 1A bis** : Graph of Balance of covariates before and after propensity score matching

Standardized mean differences are presented for each covariate before and after propensity score matching. Differences below 0.1 reflect satisfactory covariate balance, supporting the adequacy of the matching procedure in minimizing confounding bias.
