# Supplementary material for: Assessing the long-term persistence of SARS-CoV-2 in Guinea: insights from post-epidemic sentinel syndromic surveillance data
Source: Front Epidemiol. 2025 Sep 25;5:1636286. doi: 10.3389/fepid.2025.1636286 (PMC12507806; doi:10.3389/fepid.2025.1636286)
Supplement: Supplementary file 2 [file Table2.docx]

**Supplemental Material 2A:** Model Fit Indices Based on AIC and BIC

|  | **AIC** | **BIC** |
| --- | --- | --- |
| Model 1 | 395.20 | 435.10 |
| Model 2 | 394.47 | 434.38 |
| Model 3 | 392.79 | 429.06 |
| Model 4 | 391.13 | 423.78 |
| Model 5 | 389.75 | 418.77 |
| Model 6 | 388.60 | 413.99 |
| Model 7 | 387.48 | 409.25 |
| Model 8 | 387.20 | 405.33 |
| Model 9 | 387.09 | 401.60 |

**Supplemental Material 2B :** Assessment of Parsimony and Predictive Performance Across Regression Models

|  | **Resid. Df** | **Resid. Dev** | **Df** | **Deviance** | **Pr(>Chi)** |
| --- | --- | --- | --- | --- | --- |
| Model 1 | 267 | 372.20 |  |  |  |
| Model 2 | 267 | 372.48 | 0 | 0.72350 |  |
| Model 3 | 268 | 372.79 | -1 | -0.31247 | 0.5762 |
| Model 4 | 269 | 373.14 | -1 | -0.34519 | 0.5568 |
| Model 5 | 270 | 373.75 | -1 | -0.61408 | 0.4333 |
| Model 6 | 271 | 374.60 | -1 | -0.85163 | 0.3561 |
| Model 7 | 272 | 375.49 | -1 | -0.88505 | 0.3468 |
| Model 8 | 273 | 377.20 | -1 | -1.71264 | 0.1906 |
| Model 9 | 274 | 379.09 | -1 | -1.89051 | 0.1691 |

There were no significant differences between our nine models (p > 0.05). In other words, all models explained a similar amount of variance as our initial model, while being more parsimonious.
