# Supplementary figures and images for: Assessing the long-term persistence of SARS-CoV-2 in Guinea: insights from post-epidemic sentinel syndromic surveillance data
Source: Front Epidemiol. 2025 Sep 25;5:1636286. doi: 10.3389/fepid.2025.1636286 (PMC12507806; doi:10.3389/fepid.2025.1636286)

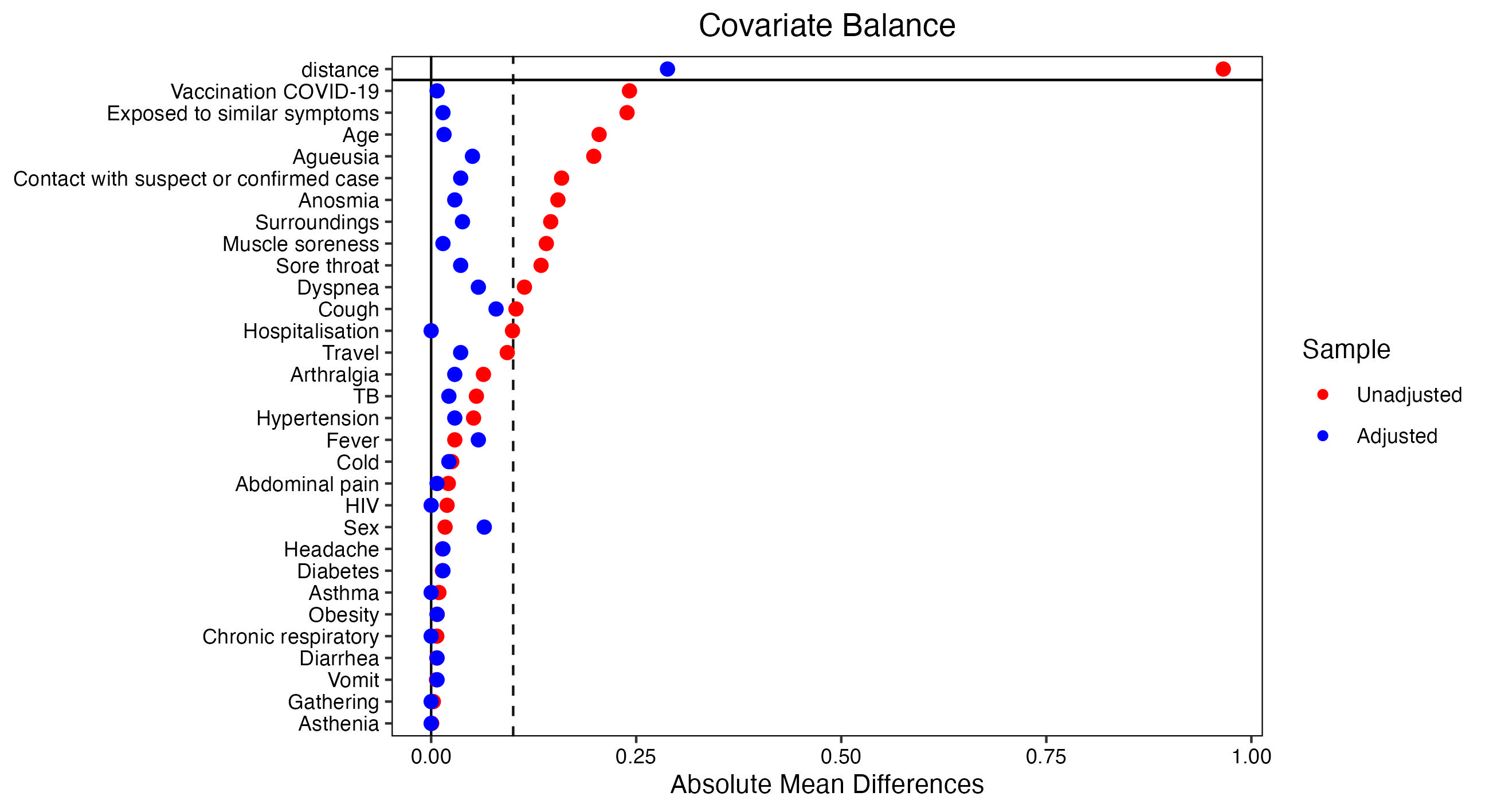

Supplement: Supplementary file 3 [file Image1.jpeg]
